# Cerebral Asymmetry of fMRI-BOLD Responses to Visual Stimulation

**DOI:** 10.1371/journal.pone.0126477

**Published:** 2015-05-18

**Authors:** Anders Hougaard, Bettina Hagström Jensen, Faisal Mohammad Amin, Egill Rostrup, Michael B. Hoffmann, Messoud Ashina

**Affiliations:** 1 Danish Headache Center and Department of Neurology, Rigshospitalet Glostrup, Faculty of Health and Medical Sciences, University of Copenhagen, Glostrup, DK-2600, Denmark; 2 Functional Imaging Unit and Department of Diagnostics, Rigshospitalet Glostrup, Faculty of Health and Medical Sciences, University of Copenhagen, Glostrup, DK-2600, Denmark; 3 Visual Processing Laboratory, Ophthalmic Department, Otto-von-Guericke-University, Magdeburg, 39106, Germany; University Of Cambridge, UNITED KINGDOM

## Abstract

Hemispheric asymmetry of a wide range of functions is a hallmark of the human brain. The visual system has traditionally been thought of as symmetrically distributed in the brain, but a growing body of evidence has challenged this view. Some highly specific visual tasks have been shown to depend on hemispheric specialization. However, the possible lateralization of cerebral responses to a simple checkerboard visual stimulation has not been a focus of previous studies. To investigate this, we performed two sessions of blood-oxygenation level dependent (BOLD) functional magnetic resonance imaging (fMRI) in 54 healthy subjects during stimulation with a black and white checkerboard visual stimulus. While carefully excluding possible non-physiological causes of left-to-right bias, we compared the activation of the left and the right cerebral hemispheres and related this to grey matter volume, handedness, age, gender, ocular dominance, interocular difference in visual acuity, as well as line-bisection performance. We found a general lateralization of cerebral activation towards the right hemisphere of early visual cortical areas and areas of higher-level visual processing, involved in visuospatial attention, especially in top-down (i.e., goal-oriented) attentional processing. This right hemisphere lateralization was partly, but not completely, explained by an increased grey matter volume in the right hemisphere of the early visual areas. Difference in activation of the superior parietal lobule was correlated with subject age, suggesting a shift towards the left hemisphere with increasing age. Our findings suggest a right-hemispheric dominance of these areas, which could lend support to the generally observed leftward visual attentional bias and to the left hemifield advantage for some visual perception tasks.

## Introduction

Hemispheric specialization has been reported for a wide range of cerebral functions [1]. Lateralization of motor control and language are well-known examples. Vision is the most widely studied and dominant sensory system in man, and highly specific visual functions, such as face, word and object recognition [2] and visuospatial processing [3] have been found to be cerebrally lateralized. However, the possible lateralization of cerebral responses to simple, non-specific visual stimulation has not been a focus of previous studies.

Visuospatial attention is dominated by the right hemisphere as evidenced by the occurrence of hemispatial neglect most often following right-sided brain lesions [4]. In addition, healthy humans show leftward bias in visuospatial tasks [5]. This phenomenon, known as ‘pseudoneglect’, suggests increased attention towards the left visual hemifield. Directing attention to a specific location results in activation enhancement in retinotopically corresponding visual cortical areas [6]. Thus, the common leftward attentional bias could correspond to an increased right hemisphere level of visual processing. Other factors could produce interhemispheric asymmetry of cortical visual function: monocular stimulation causes relatively larger responses in the contralateral hemisphere [7] and stimulation of the dominant eye produces greater responses than stimulation of the non-dominant eye [8]. Therefore, ocular dominance and interocular visual acuity differences may also play a role in cerebral lateralization. In addition, the visual cortical areas are anatomically asymmetric with a larger grey matter volume around the right calcarine sulcus [9]. Cerebral lateralization often depend on age, gender and handedness [1,9], which could play a role in lateralization of visual function. In the present study, we used high-field functional magnetic resonance imaging (fMRI) to examine cerebral lateralization of the visual system. The subjects were presented binocularly with a symmetric visual stimulus (a black and white dartboard pattern) designed to activate large expanses of visual cortex. The objective of the study was to assess differences in visual activation between the two hemispheres by voxel-wise comparison while investigating the relation of these possible interhemispheric differences to handedness, interocular difference in visual acuity, ocular dominance, horizontal line-bisection performance, age, gender, and voxel-wise grey matter volume differences.

## Materials and Methods

### Subjects

We recruited 56 healthy volunteers (36 F, 20 M, mean age 35.4 years [range 19–61]). See Table 1 for subject characteristics. All had their medical history taken and underwent a general and neurological examination. Exclusion criteria were: Any history of neurological disease, including migraine and frequent tension-type headache, any ophthalmologic disorders except refractive anomalies, any serious somatic or psychiatric conditions, or daily intake of medication with effect on the central nervous system. Some of the volunteers served as healthy controls in a previously published study of migraine [10].

**Table 1:** Characteristics of study population (N = 54). Data are presented as mean (SD or range). R: Right, L: Left.

Age (years)	35.4 (19 to 61)
Male/female	19/35
Weight (kg)	73.2 (14.7)
Visual Acuity (R; L)	R: 0.9 (0.5); L: 0.9 (0.5)
Visual Acuity, Difference (R-L)	-0.034 (0.34)
Ocular Dominance (n)	R: 35, L: 19
Edinburgh Handedness Inventory (-100 to 100, left is negative)	83.0 (47)
Line Bisection (mm deviation from true center, leftwards is negative)	-1.3 (2.5)

Prior to scanning, handedness was assessed using the Edinburgh Handedness Inventory [11]. Decimal visual acuity was determined with the Freiburg Acuity and Contrast Test (FrACT) [12], i.e. Landolt-C optotypes displayed on an LED display (22 inch Brilliance 220SW LED display, 1680 x 1050 pixels; Philips, Best, The Netherlands) at a viewing distance was 3 meters. In order to assess if interocular differences in visual acuity influence interhemispheric response differences, we deliberately assessed the uncorrected visual acuity of each eye, and refractive error was not corrected for in the MRI stimulation system. All subjects reported normal or corrected to normal vision and none had any ophthalmological disorders apart from minor refractive error. Thus, all of the subjects were able to see the simple stimulus used, but some of the subjects did not see the stimulus equally well with both eyes, which was what we intended, as we wanted to relate this effect to the interhemispheric difference in response to the stimulus.

Eye dominance was examined using the ‘hole in hand’ variant of the Miles test [13]. In order to estimate visual attentional bias, all subjects performed a horizontal line bisection test [5] in which they were instructed to mark the midpoint of 18 cm long black lines presented on 10 consecutive sheets of white A4 format paper. The average deviation from the true midpoint was calculated for each subject.

A table of subject age, gender, handedness, ocular dominance, visual acuity difference, and line-bisection performance is available as supporting information (S1 Dataset).

The Ethics Committee of the County of Copenhagen (H-KA-20060083) approved the study, which was undertaken in accordance with the Helsinki Declaration of 1964, as revised in 2008. The study was carried out at Glostrup Hospital, Copenhagen, Denmark from April 2011 to July 2013. All subjects gave written informed consent to participate in the study. The study protocol was not made publicly available before study initiation.

### MRI procedure

MRI was performed on a 3.0T Philips Intera Achieva scanner (Philips Medical Systems, Best, The Netherlands) using a 32-element phased-array receive head coil. Anatomical images were acquired using a T1-weighted three-dimensional turbo field-echo sequence (170 sagittal slices of 1 mm thickness; in-plane resolution 1 x 1 mm; repetition time 9.9 s; echo time 4.6 ms; and flip angle 81 deg).

Functional imaging used a gradient-echo echo-planar imaging sequence (32 slices of 4.0 mm thickness; slice gap 0.1 mm; field of view 230 x 230 mm; in-plane acquired resolution 2.9 x 2.9 mm; repetition time 3.0 s; echo time 35 ms; flip angle 90 degrees; and SENSE (SENSitivity Encoding) factor 2). Phase encoding was carried out in the anterior-posterior direction to avoid left-right asymmetry. Dummy scans (two volumes) were applied to ensure steady-state longitudinal magnetization. The lighting conditions inside the scanner and in the scanner room were identical in each scan session.

**Fig 1:**
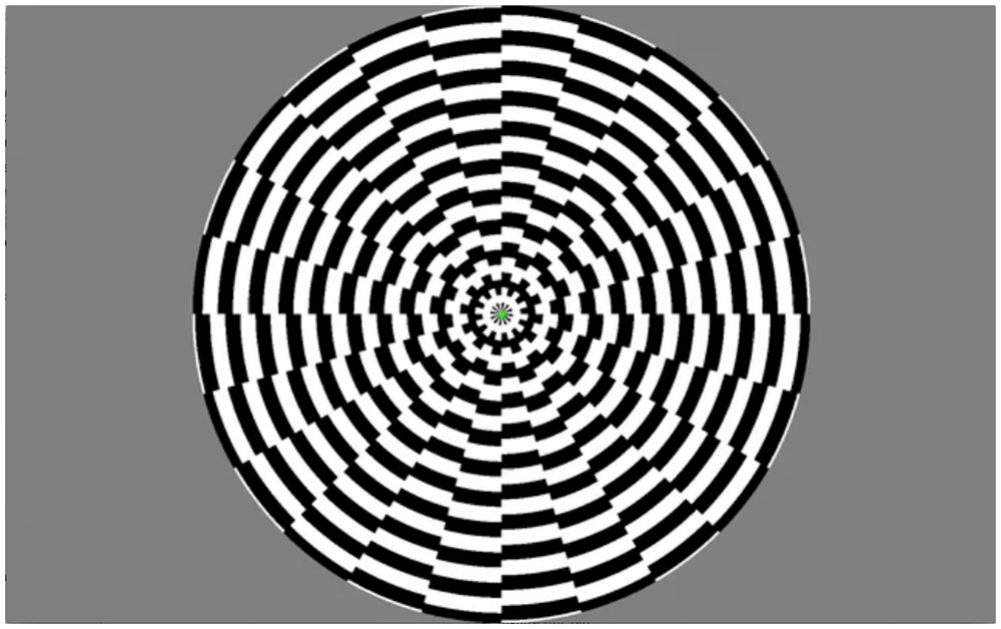
A screenshot of the applied visual stimulus.

Visual stimulation was presented binocularly using OLED video goggles (NordicNeuroLab, Bergen, Norway; SVGA, 800 x 600 pixels, refresh rate 85 Hz, FOV 30 horizontal, 23 vertical, stimulus luminance: 70–110 cd/m^2^). A fiber optic cable connected the system to a control computer outside the scanner room. The block-design stimulation paradigm consisted of an alternation of stimulation and rest blocks each comprising 18 s. A symmetric full-field high contrast (for the applied video goggles this was ≥100:1 Intrinsic (Measured per VESA FPDM Standard) motion stimulus was used to drive large expanses of visual cortex [14], i.e., a moving black and white dartboard pattern [diameter: 22 deg (circular aperture); ring width: 0.6 deg; spoke width: 15 deg; patterns in each spoke moved in opposite directions, alternately inward and outward, with random changes of the motion direction approx. every 2–3 s]. A screenshot of the stimulus is shown in Fig 1. The purpose of this stimulus was to activate cortical areas involved in visual processing. The aim of the study was to investigate interhemispheric differences of such general visual processing and relate the differences to handedness, interocular difference in visual acuity, ocular dominance, age, gender, and anatomical differences. Therefore we did not use tasks specifically involving visuospatial attention or other specialized functions. The stimulus was generated using freely available Matlab-based software (http://vistalab.stanford.edu/software). A complete scan comprised thirty-two 18 s blocks and lasted 576 s. The subjects were instructed to fixate on a central fixation point during the entire scan. They performed no additional stimulus driven task (e.g. button press) to avoid task related effects on response lateralization [15]. The onset of visual stimulation was triggered by the scan acquisition. Following stimulation the subjects were asked if they were able to stay focused and awake during the stimulation. Subjects who reported that they did not, or whose BOLD signal response indicated that they fell asleep, were excluded leaving 55 out of 56 subjects for the further analyses.

All subjects underwent one scan with the video goggles in the standard position and one scan with the goggles rotated 180 degrees to control for lateralization effects caused by asymmetry of the stimulation. The sequence of goggle positions was changed every time a new subject was scanned to avoid differences between scans obtained in the two positions to be due to time effects.

### Data analysis

The main objective of the analysis was to compare functional activation in response to visual stimulation between left and right cerebral hemispheres. This was achieved by direct comparison of voxels in left hemispheres to corresponding voxels in right hemispheres by comparing fMRI data in the radiological convention to mirrored data (i.e. flipped horizontally in the left-right direction).

Analysis of the fMRI images to identify regions exhibiting significant stimulus-correlated changes in blood oxygen level dependent (BOLD) signal was carried out in a multi-stage process using FMRIB's Software Library (FSL) ver. 4.1.19 [16]. To validate this analysis method we conducted an experiment using half-sided hemifield stimulation in five subjects (see below).

Tests for correlation between the measured parameters (i.e. handedness, interocular difference in visual acuity, ocular dominance, line-bisection performance, age, and gender) was performed by calculation of Kendall’s tau rank correlation coefficient. Thus, a total of 15 correlation tests were carried out. The tests were not corrected for multiple testing. Statistical calculations were carried out using R ver. 2.14.1 for MacOS X.

### First-level voxel-wise analysis

The following data were available from each subject: functional data from scan with goggles in standard position, functional data from scan with goggles flipped 180 degrees and a 3D anatomical scan. Mirror images of the acquired functional and anatomical data were created for each subject. In order to avoid left-right bias from registration to an asymmetric standard space, a symmetric version of the Montreal Neurological Institute (MNI) 152 template was created by adding a mirrored version of the template to the original version. This approach is commonly used for interhemispheric comparison [17,18]. Functional data were registered to the brain extracted T1-weighted high-resolution scans and to the symmetric MNI152 template using FSL FLIRT linear registration. Registration from high resolution structural to standard space was further refined using FSL FNIRT nonlinear registration. Mirrored functional images were registered to the corresponding mirrored T1-weighted images.

First-level analysis was carried out using FSL FEAT (FMRI EXPERT Analysis Tool) ver. 5.98. Pre-processing included slice time correction, spatial smoothing (FWHM 5 mm), high pass filtering (cut-off 36 s), head motion correction using FSL MCFLIRT and brain extraction of functional and anatomical images using FSL BET (Brain Extraction Tool).

After pre-processing, a voxel-based analysis was performed using a general linear modeling approach of seven regressors (main stimulus (box-car), a temporal derivative and six motion regressors). The main stimulus box-car function was convolved with a canonical single gamma hemodynamic response function. Mean activation and deactivation maps of all scan sessions from eligible subjects was calculated using a fixed-effects analysis in FSL FEAT.

### Validation experiment

**Fig 2:**
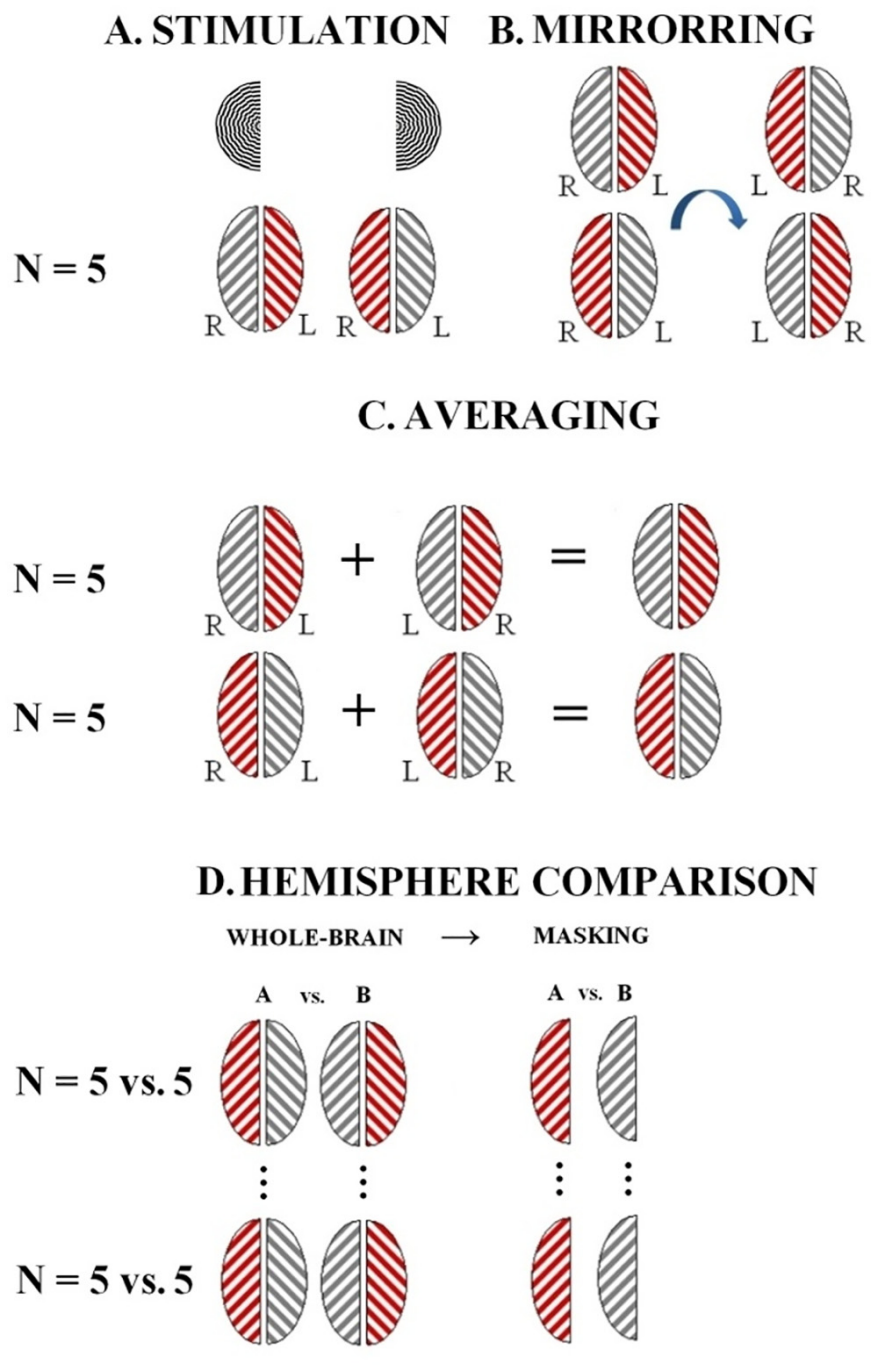
Methods of the validation experiment comparing “activated” hemispheres (contralateral to the stimulation) to the opposite “non-activated” hemispheres. **(a)** Two scan sessions were carried out in 5 subjects out during visual stimulation of the left and right hemifield, respectively. **(b)** Left-right mirrored copies of the acquired data were created for each scan session. **(c)** After processing the first-level fMRI results, left hemifield stimulation images were averaged with mirrored right hemifield images and vice versa. Thus,”activated” hemispheres, contralateral to the stimulation, are averaged in the same side of the image space. **(d)** Hemispheres were compared in a higher-level analysis. A paired analysis of brains with the”activated” hemispheres in the right side of image space vs. corresponding mirrored images was carried out. This would produce the same results (with opposite sign) for both hemispheres. To avoid this redundancy the left side of the image space was zeroed by a binary mask.

To validate the method of functional interhemispheric comparison, we conducted an additional experiment in which subjects were stimulated in one visual hemifield at a time, to compare “activated” hemispheres (contralateral to the stimulation) to the opposite “non-activated” hemispheres (Fig 2). This experiment was done to assess the effects of data flipping, averaging and registration to a symmetric standard space. Specifically for this experiment, we included five healthy volunteers (1 F, 4 M, mean age 29.6 years [range 24–37 years]). Each subject was scanned twice using the procedure and visual stimulation described above, but with unilateral stimulation: one scan with stimulation in the left hemifield and one scan with stimulation in the right hemifield.

After processing the first-level fMRI results, left-hemifield stimulation images were averaged with mirrored right-hemifield stimulation images and vice versa using a fixed-effects analysis in FSL FEAT (Fig 2C). Thus,”activated” hemispheres, contralateral to the stimulation, were averaged in the same side of the image space. A paired analysis of brains with the”activated” hemispheres in the right side of image space vs. corresponding mirrored images was carried out using FSL’s FMRIB’s Local Analysis of Mixed Effects (FSL FLAME). The null-hypothesis was no average difference between “activated hemisphere” and “non-activated hemisphere”. Z statistic images were thresholded using clusters determined by Z > 2.3 and a corrected cluster significance threshold of P < 0.05 (using a distribution based on Gaussian Random Field Theory). This analysis would produce the same results (with opposite sign) for both hemispheres. To avoid this redundancy the left side of the image space was zeroed by a binary mask. For the method to be considered valid this should show activation differences in relevant brain areas for the contrast “activated hemisphere” > “non-activated hemisphere” and no differences for “non-activated hemispheres” > “activated hemispheres”.

### Goggle position

We assessed if the goggle position *per se* would cause differences in the activation pattern following full-field visual stimulation. A second-level paired voxel-wise group comparison was carried out using FSL FLAME (goggles in the standard position vs. goggles rotated 180 degrees).

### Interhemispheric comparison

**Fig 3:**
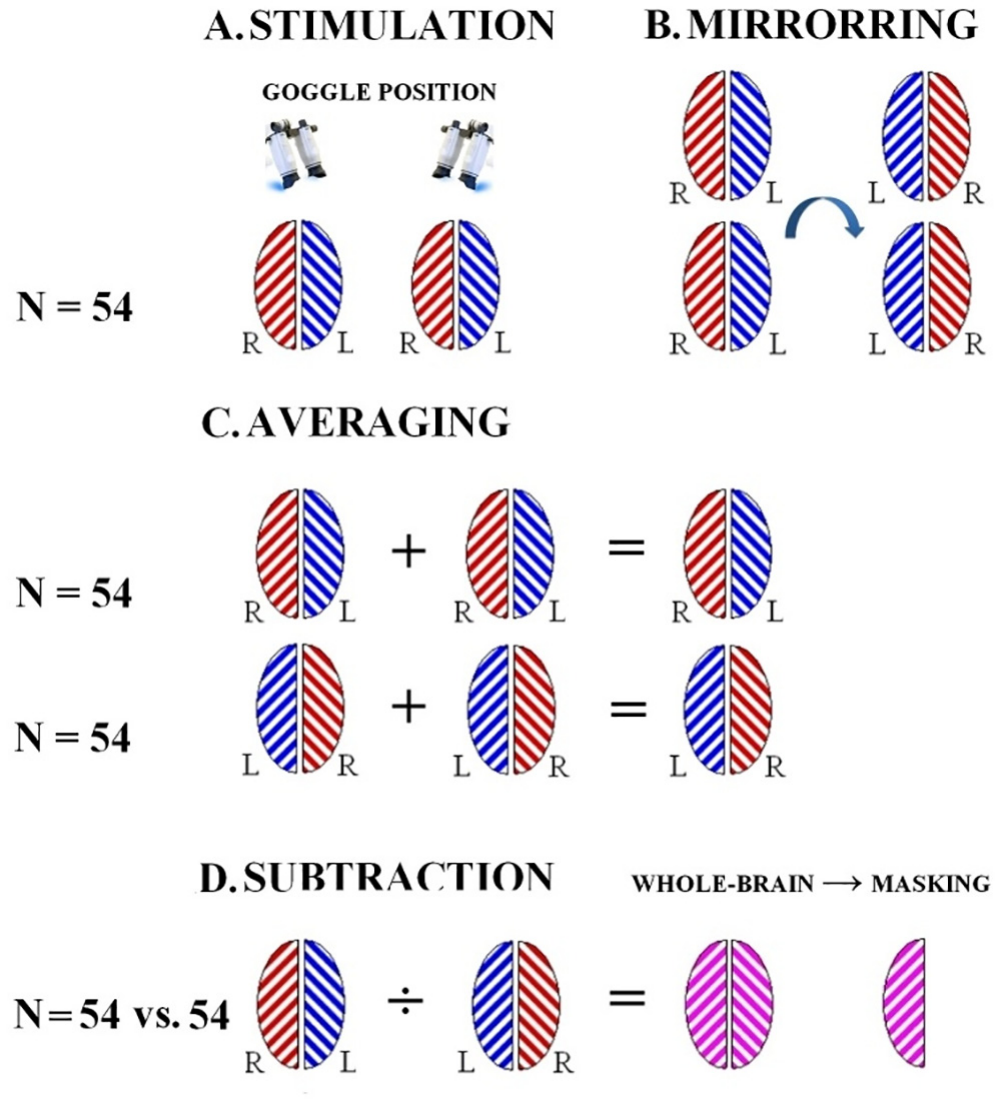
Methods of the main experiment comparing activation of left hemispheres to right hemispheres. **(a)** Two scan sessions were carried out in 54 subjects out during full-field visual stimulation: one with the stimulation goggles in the standard position and one with the goggles flipped 180 degrees. **(b)** Left-right mirrored copies of the acquired data were created for each scan session. **(c)** After processing the first-level fMRI results, data from the two scan sessions were averaged for each subject. **(d)** First-level results of mirrored data were subtracted from first-level results of data in the original orientation to produce left-right difference images that would allow for adding covariates to the model. The difference images have the same values in the right and left sides of image space but with opposite signs. To avoid this redundancy the left side of the image space was zeroed by a binary mask.

To compare left and right hemispheres, data in the original orientation were compared to mirrored data (Fig 3B). Functional data from the two goggle positions were averaged for each subject (Fig 3C). Followingly, left-right difference maps were calculated by subtracting first-level results of mirrored data from first-level results of original data in a fixed-effects analysis (Fig 3D). To investigate the sources of interhemispheric differences, we analyzed left-right difference maps from each subject in a general linear model with seven subject-dependent regressors: mean effect, handedness, interocular difference in visual acuity, ocular dominance, line-bisection performance, age, gender, and one voxel-dependent regressor (anatomical grey matter volume maps created using the FSL script feat_gm_prepare [19]). The latter integrates a voxel-based morphometry grey matter map (difference between right and left hemisphere) into the analysis to correct for functional activation differences due to differences in grey matter volume. The analysis was performed using FSL FLAME. As in the first-level analysis, Z statistic images were thresholded using clusters determined by Z > 2.3 and a corrected cluster significance threshold of P < 0.05.

### Region of interest-based analysis

In order to assess the absolute level of activation in areas of interhemispheric differences (i.e. to see if differences are due to activation or deactivation) we carried out a secondary region of interest (ROI) analysis. The ROIs used were binary masks of the areas that we found to be different in response magnitude between left and right hemispheres in the voxel-wise analysis.

Mean percentage signal changes during activation were extracted from first-level FSL FEAT results using Featquery (a part of the FSL software package). To exclude differences caused by erroneous registration, values were extracted from functional data registered to the standard (asymmetric) MNI152 2mm template. Thus, the ROI-based analysis allowed us to evaluate if differences were artifacts from registration to a symmetric template.

Laterality indices were calculated for each pair of ROI as the difference between the mean left-side ROI value and the mean right-side ROI value, divided by the sum of these two values, thus allowing laterality index values to range from -1 (extreme right lateralization) to 1 (extreme left lateralization).

## Results

All subjects completed the study. One subject was excluded because she fell asleep during one scan session. All other subjects complied well with the study procedures. The T1-weighted images were reviewed by an experienced neuroradiologist who found a structural abnormality in one subject, causing this subject to be excluded. Thus, eligible datasets were available in 54 out of 56 subjects.

**Fig 4:**
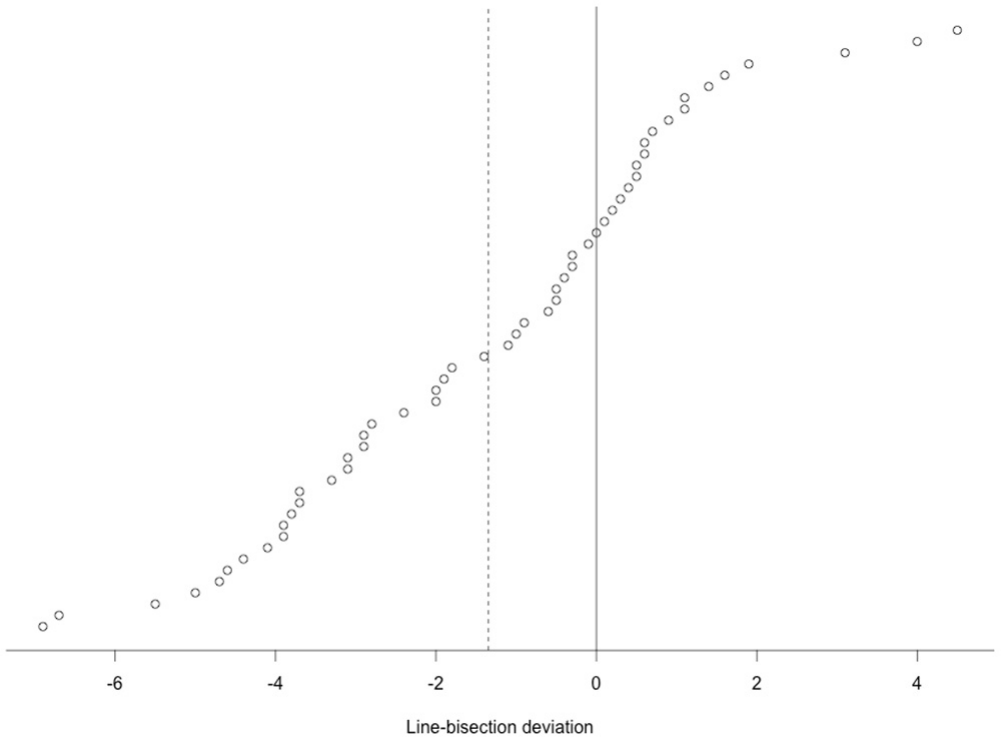
Distribution plot of the line-bisection results, i.e. the deviation for each subject in mm. Subjects are ordered by their linc-bisection performance value. The dashed line marks the average deviation.

**Fig 5:**
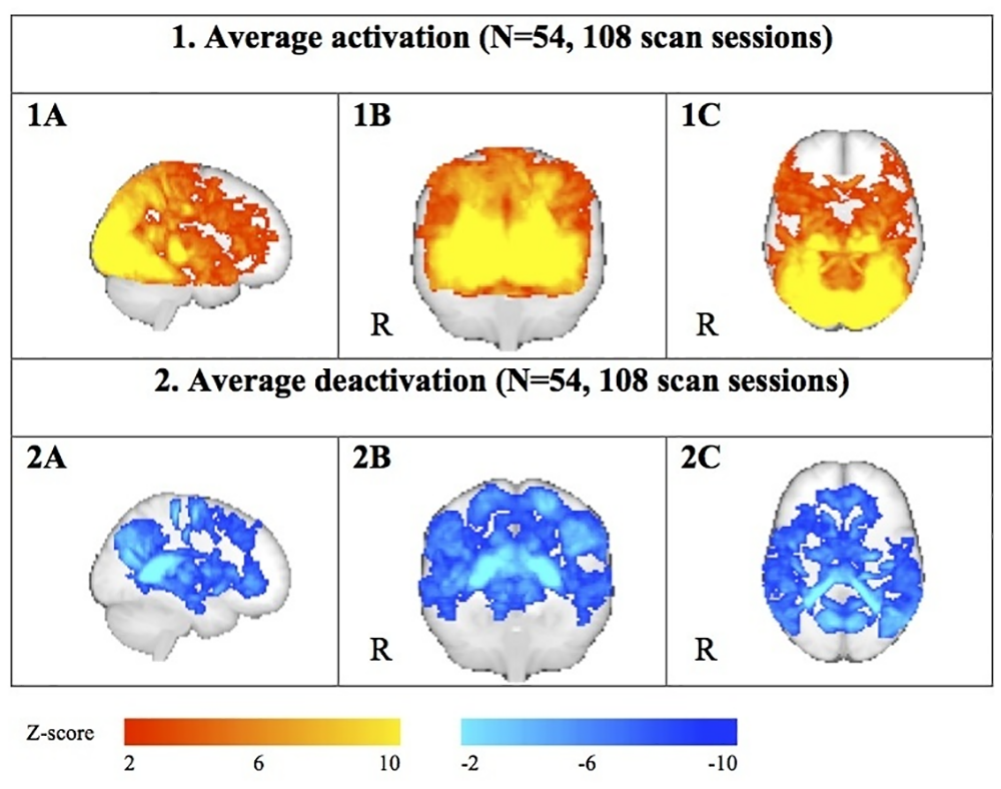
Maximum intensity projections of the average activation and deactivation for all scan sessions (2 sessions with 2 different goggle positions in all eligible subjects). Due to the high power of the approach great expanses are activated, which creates the impression that activation and deactivation appear to overlap locally. Both maps are, however, exclusive.

Additionally acquired data from the subjects are summarized in Table 1. Consistent with previous studies, the subjects deviated toward the left in the line bisection test at a group level (mean deviation = 1.35 mm, 95% CI: [0.7–2.04], *t*_53_ = 3.9, *P* = 0.0003). A distribution plot of the line-bisection results is shown in Fig 4. Tests for correlation between the parameters showed a significant correlation only between line-bisection performance and interocular differences in visual acuity (Kendall’s *tau* = -0.23, *P* = 0.017). The average activation and deactivation to the visual stimulation is shown in Fig 5.

### Goggle comparison

The whole-brain comparison of functional scans acquired during the two different goggle positions showed no difference in activation, indicating no left-right asymmetry of the stimulation setup.

### Validation experiment

**Fig 6:**
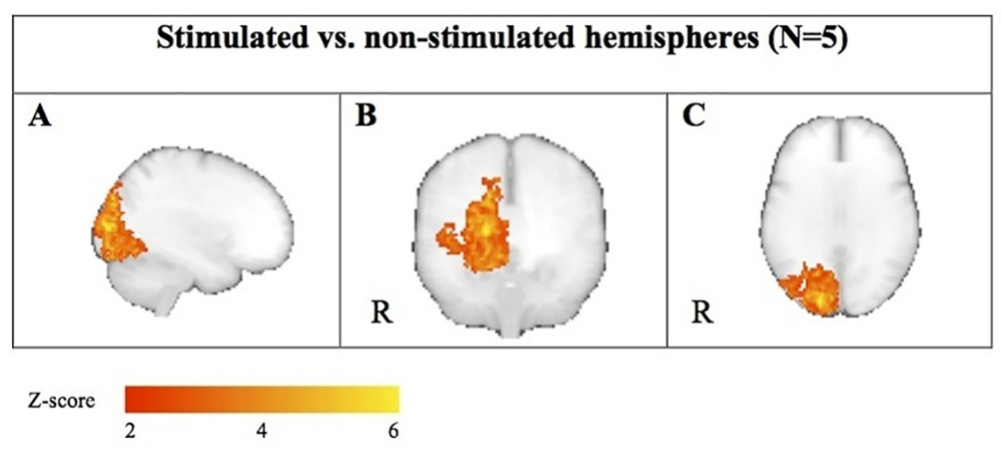
Maximum intensity projections (“glass-brain”) presentations of the voxel-wise results from the validation experiment. Results for “activated” > “non-activated” hemispheres in five subjects are shown on the right hemisphere side (marked with an R), a marked activation in the occipital lobe, peaking in the primary visual cortex, while results for “non-activated” > “activated” hemispheres, depicted on the left hemisphere side show no difference.

Comparing “activated” to “non-activated” hemispheres in five subjects showed a marked activation in the occipital lobe, peaking in the primary visual cortex (MNI coordinates (X, Y, Z) = (-14, -94, 8)) for “activated” > “non-activated” hemispheres and no difference for “non-activated” > “activated” hemispheres. See Fig 6. The applied method is thus useful for hemisphere comparison.

### Interhemispheric differences

**Fig 7:**
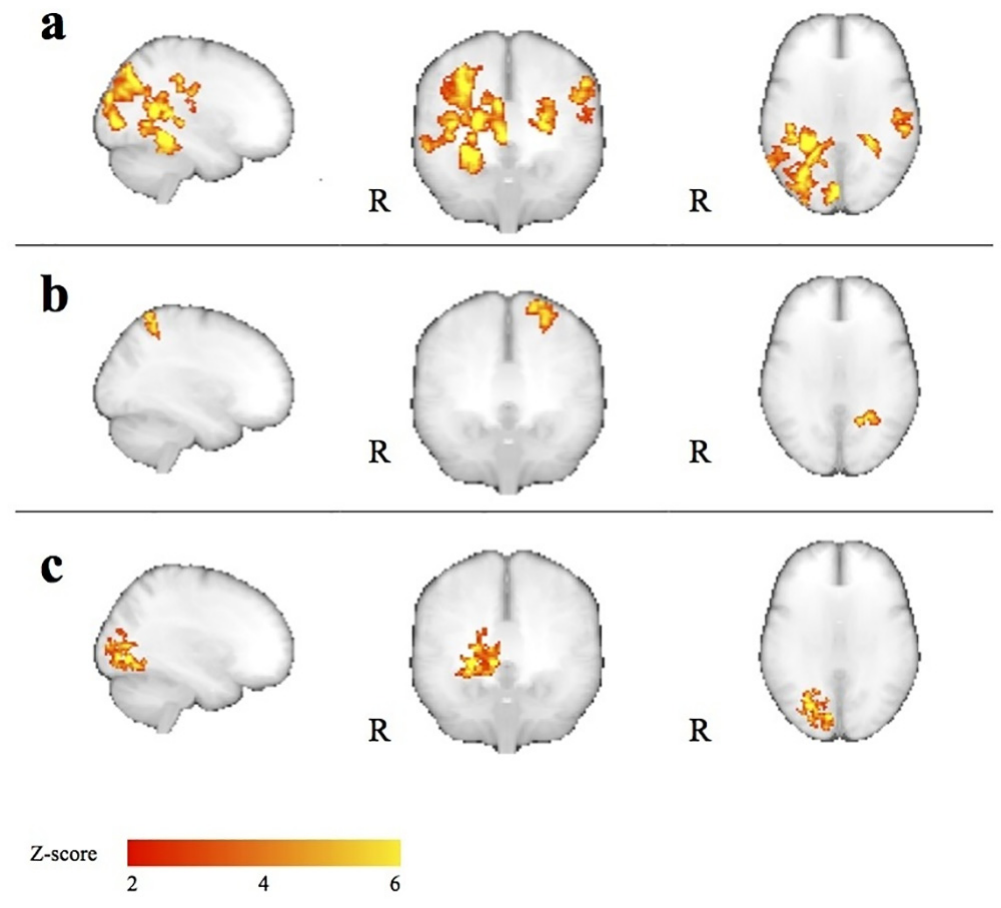
Maximum intensity projections (“glass-brain”) presentations of the voxel-wise results from the interhemispheric comparison analyses. Right>Left activation is shown on the right hemisphere side (marked “R”) while Left>Right activation is shown on the left side. **(a)** Left-to-right activation differences corrected for six different parameters (See text). **(b)** and **(c)** Differences that correlate positively with age and grey matter volume, respectively.

The voxel-wise analysis revealed multiple areas of hemispherically lateralized activation (see Table 2 and Fig 7). Handedness, difference in visual acuity, line-bisection performance and gender did not influence the results in the voxel-wise analysis. Increased activation of areas in and around the calcarine sulcus in the right hemisphere was due to a relatively larger grey matter volume of these areas in the right hemisphere. The activation in one area of the left hemisphere positively correlated with subject age, i.e. higher activation in the superior parietal lobule of the left hemisphere was seen in subjects of higher age (cluster-wise P = 0.012, maximum Z-value = 3.73, MNI coordinates (X,Y,Z) = (-26,-52,66)).

**Table 2:** Significant voxel clusters of left-right interhemispheric differences from the voxel-wise general linear model analysis. Also shown are results from the co-variates “Age” and “Grey matter volume”. Voxels: number of voxels in significant cluster. Z-max: maximum Z value of most significant voxel. X, Y, Z: Montreal Neurological Institute coordinates of most significant voxel. LI: Laterality Index based on the region-of-interest analysis, see Methods. NA: Not applicable.

Cluster no.	Voxels	P-value	Z-max	X	Y	Z	Location	LI
**Right>Left**								
1	942	0.00000000627	4.6	-32	-70	40	Superior/inferior parietal lobule	-0.16
2	884	0.0000000165	5.8	-30	-44	-12	Temporal fusiform cortex/Precuneus	-0.34
3	416	0.0000115	4.57	-6	-84	2	Visual cortex V1/V2	-0.16
4	173	0.045	3.6	-60	-62	-6	Middle temporal gyrus, temporooccipital part	-0.16
5	172	0.047	4.72	-42	-38	10	Posterior supramarginal gyrus	-0.23
**Left > Right**								
6	319	0.001	4.02	-56	-30	40	Inferior parietal lobule	0.15
7	288	0.0022	4.44	-26	-44	16	Anterior intraparietal sulcus	0.13
**Age**								
8	240	0.00073	3.93	-18	-56	68	Superior parietal lobule	NA
**Grey matter volume**								
9	511	0.0000151	5.76	-26	-76	-4	Visual areas V1, V2, V3 and V4	NA

### Region of interest-based analysis

**Fig 8:**
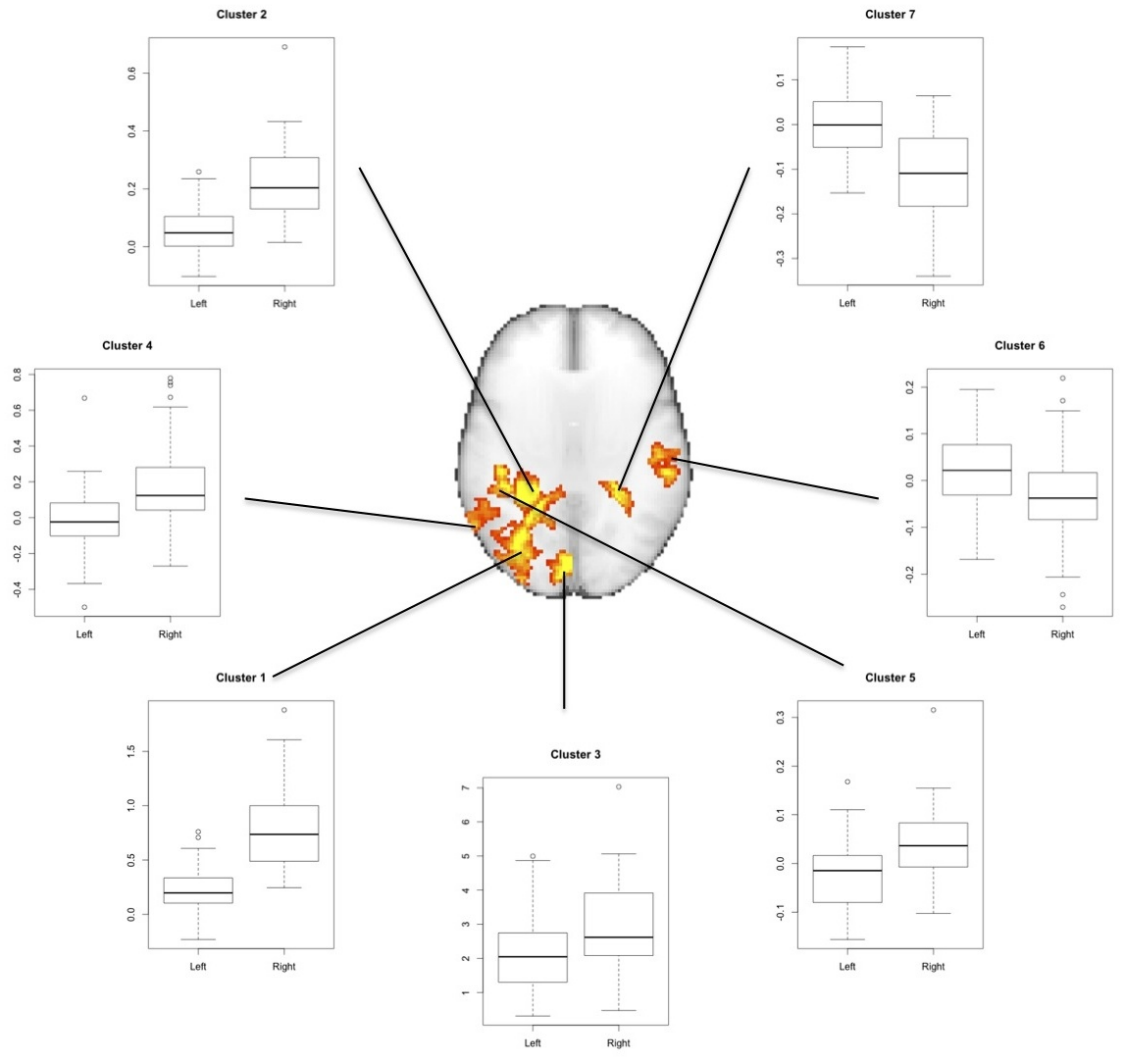
Region of interest-based analysis showing absolute BOLD responses to visual stimulation in areas of lateralized activation from the voxel-wise analysis. Cluster numbers are given in Table 2. Values on the y-axes of the boxplots are mean percentage BOLD signal changes.

Since the observed hemisphere differences could be due to systematic error of alignment to the symmetrical standard space, we carried out a secondary ROI analysis. The areas that were found to be different between hemispheres were used as ROIs. Using these ROIs we extracted the mean percentage activation levels extracted from the original, unflipped data registered to the normal, asymmetric standard space (Fig 8). Laterality indices are given in Table 2.

These findings indicate that the functional asymmetries from the voxel-wise analysis were not due to image flipping or registration to a symmetric template. Interestingly, areas of interhemispheric differences favoring the right side were generally due to a larger right than left activation, while areas favoring the left side generally reflected right hemisphere deactivation.

## Discussion

We conducted the first fMRI-BOLD study of hemispheric lateralization of visually driven activation in the human brain, applying a method of “brain flipping” that allows for direct voxel-for-voxel comparison of hemispheres. This approach has been used for interhemispheric comparison in several previous studies [17,18,20]. Initially, we successfully validated this method for analysis of responses to visual stimulation specifically.

Lateralized activation was observed in several cortical areas, but most evidently, the primary and secondary visual cortices and areas of higher-level visual processing in the parietal lobules and the temporal gyrus were lateralized to the right hemisphere. This was partly, but not completely, explained by an increased grey matter volume in the right hemisphere of the early visual areas. Interestingly, difference in activation of the superior parietal lobule (SPL) was correlated with subject age, and thus may shift towards the left hemisphere with increasing age.

Behavioral studies have shown that series of rapidly presented visual stimuli are better identified when presented in the left than in the right hemifield [21] and that subjects tend to choose the stimulus whose salient feature is on the left-hand side more than the stimulus with a salient feature on the right-hand side [21–23]. In addition, studies on participants with central scotoma (< 6° diameter), either a physiological scotopic central scotoma or due macular diseases, indicate a superiority of the left hemifield. They shift their fixation point preferentially, in approx. 75% of the cases, into their left or left inferior visual field [24], leaving only 12% for the other quadrants. These findings support hemispheric specialization of cortical visual function and further suggest a preponderance of lateralization to the right hemisphere. In support of this, a recent study found that the threshold for eliciting visual perception is significantly lower in the right hemisphere compared to the left hemisphere when applying direct electrical stimulation to the cortex of awake subjects, suggesting increased neuronal excitability [25]. Interestingly, this effect was observed in almost the exact same areas where we observed right hemisphere dominance in the present study.

The commonly observed left-ward visual attentional bias has also been related to a right-hemisphere dominance. An MR tractography study showed a correlation between the laterality of white matter tracts and horizontal line bisection performance [26], demonstrating increased tract size in the right hemisphere of most subjects. This would suggest an anatomical reason for the increased right hemisphere neuronal responses, related to line-bisection performance observed in the present study. A study using intraoperative electrical stimulation in awake subjects showed that stimulation of the right inferior parietal lobule or the right superior temporal gyrus caused rightward shifts in line-bisection deviation, supporting that visuospatial attentional bias is governed by a right-hemisphere superiority [27]. Judgement of whether horizontal lines were correctly prebisected during fMRI acquisition showed activation predominantly in the right SPL and IPL as well as the cerebellum [28,29].

### Visual function of lateralized areas

In the present study, we found hemispheric response lateralization of several visual areas. The potential implications for visual function are not obvious for all of these areas. However, they are likely to be related to differential processing of information in the left and right hemifield, as many visual areas have been demonstrated to comprise a retinotopic map of the contralateral visual hemifield [30]. This is particularly clear for the early, but also evident for higher-level visual areas.

While we found no relation between line-bisection performance and interhemispheric differences, the observed right-hemisphere lateralization may at least partly be due to increased attention towards the left hemifield, especially as attentional effects have been shown to modulate the BOLD response in a localised and even retinotopic manner [6]. In this context, the fact that the right-hemisphere lateralization affected areas of the parietal lobes, i.e. SPL, IPL, and precuneus, might be of importance. The parietal lobes generally function as association areas, i.e. they are involved in integrating, storing and processing a wide variety of stimuli, including a vast amount of visual information [31]. Here, the SPL, inferior parietal lobule (IPL), and precuneus have specific roles in visuospatial processing and visual attention. The SPL is part of the dorsal processing stream of visual information also known as the “where” stream, which mainly functions in localization and visuomotor integration [32]. Specifically, the SPL is involved in visuomotor control, e.g. visual reaching [31,33], and spatial shifting of attention [34]. Lesions to this area often result in optic ataxia [31]. The IPL, situated between the SPL and the temporal lobe, have been linked to different attention related symptoms including spatial neglect, optic ataxia, as well as deficits in both maintaining attention and also responding to changing events [35–37]. These findings are generally functions of the right IPL, whereas the role of the left IPL is less clear. The precuneus is specifically activated during certain spatial tasks including voluntary attention shifts and during visuospatial mental operations [38].

### Relevance of lateralization of visual function

Previous studies have suggested two right-lateralized networks underlying visuo-spatial attention: the dorsal frontoparietal network (DFN), which is active during voluntary (“top-down” attention), and the ventral frontoparietal network (VFN), which is used to direct attention to salient events (“bottom- up” attention) [39]. The right-lateralized higher-level areas in our study represent the posterior part of the DFN (posterior parietal cortex, middle temporal, fusiform cortex), while we found no stimulus-driven activation of the VFN (temporo-parietal junction, inferior/middle frontal gyrus, frontal operculum) and even a deactivation of the right aIPS.

The syndrome of visuospatial neglect is characterized by a deficit in attention to and awareness of one side of space. It is most commonly seen following lesions to the right hemisphere, causing left neglect, but right visuospatial neglect in patients with left hemisphere damage also occurs [40]. The key brain regions associated with neglect are the IPL [41,42], the superior temporal gyrus [43], the inferior medial temporal lobe [42]. We did not find a significant correlation between line-bisection performance and interhemispheric activation difference of the cortical areas that were functionally lateralized to the right hemisphere. However, it is plausible that other attentional mechanisms, likely involved in top-down processing, account for these interhemispheric differences and that that these may relate to subtle bias towards the left hemifield in other visuospatial tasks. Further studies are needed to clarify the full consequences of right hemisphere lateralization of visual processing.

### Limitations of the study

To reduce bias, we ensured the symmetry of the stimulus by flipping the video goggles and we took into account several physiological parameters that could produce left-to-right asymmetry (handedness, visual acuity, ocular dominance and visual attention). Asymmetry caused by the MRI acquisition cannot be entirely ruled out. Phase encoding of the echo planar imaging sequence was carried out in the anterior-posterior direction to avoid left-right bias (see Methods). Theoretically, the scanner could also cause stimulus asymmetry by distorting image quality in the video goggles, but this effect seems unlikely. Thus, we believe that the interhemispheric differences reported here represent true physiological effects and not artifacts due to technical issues. However, a replication of the study using a different hardware setup, i.e. a different scanner and stimulation equipment, would be ideal.

The subjects performed the line bisection with their dominant hand, i.e. 3 out of 54 subjects used their left hand. Both handedness and the actual hand used modulates line-bisection performance. Dextrals err farther to the left than sinistrals, while use of the left hand causes larger leftward error than when using the right hand [5]. Thus, this would have only a minor effect on the line-bisection results.

Subjects were not selected based on handedness, and as consequence the vast majority were strictly right-handed (EHI = 100 for N = 35 subjects). This may explain why we did not find an effect of this parameter on the cerebral lateralization of activation.

The stimulus used was developed for general visual activation of large expanses of the cerebral visual system without selectively activating specific visual functions. Indeed, this effect is evident from the mean activation pattern produced by the stimulus in this study (Fig 1). However, it cannot be fully excluded that the observed lateralization of activation could be to some extent specific for this particular stimulation, i.e. a high-contrast black and white circular pattern with random motion. Eye movements were not monitored during scanning. Eye movements could theoretically cause some of the observed lateralization, but it would require the 54 subjects to systematically deviate their eyes in the same way, which is unlikely given the confirmed symmetry of the stimulation.

### Conclusion

We observed interhemispheric differences in visual activation by a black and white checkerboard stimulus in a large group analysis of neurologically and ophthalmologically healthy human subjects. The lateralization was caused by either activation or deactivation of the right hemisphere. We found some of this lateralization to be dependent on age and increased right hemisphere grey matter volume, but not on line-bisection deviation, handedness, gender, ocular dominance or visual acuity.

Our findings suggest a right-hemispheric dominance of these areas, which could lend support to the generally observed leftward visual attentional bias and to the left hemifield advantage for some visual perception tasks. These observations spur further investigations of the consequences of this asymmetry to the understanding of human visual function and attentional mechanisms.

## Supporting Information

S1 Dataset Subject characteristics. Age, gender, Edinburgh Handedness Inventory, ocular dominance, interocular visual acuity difference, and line-bisection test performance for each subject.
(XLS)
